# Progress and new questions in understanding the mechanisms controlling plant nitrogen responses

**DOI:** 10.5511/plantbiotechnology.25.1105a

**Published:** 2026-03-25

**Authors:** Shuichi Yanagisawa

**Affiliations:** 1 Agro-Biotechnology Research Center, Graduate School of Agricultural and Life Sciences, The University of Tokyo,1-1-1 Yayoi, Bunkyo-ku, Tokyo 113-8657, Japan

**Keywords:** nitrate signaling, nitrogen use efficiency, transcription factor, transcriptional cascade, transcriptional regulation

## Abstract

Plants require larger quantities of nitrogen (N) than of other soil nutrients, making N availability critical for plant growth and crop yield. However, levels of N sources available in soils vary across different regions of the world, but are often insufficient to support optimum plant growth. In addition, N availability fluctuates both temporally and spatially. Therefore, plants must sense and respond to environmental variations in N levels or to N shortages. Nitrate is both the primary source of N for plants and a critical signaling molecule that regulates N assimilation and many other physiological processes in plants. Recent studies have unraveled the mechanism of nitrate signaling and placed it at the center of the regulatory pathways governing plant responses to N availability. This review briefly summarizes the recent advances that revealed the mechanisms controlling plant responses to N availability. It focuses particularly on the mechanism of nitrate signaling, before addressing the new questions that have emerged from recent findings. Finally, it discusses how recent insights into the mechanisms regulating plant responses to N availability can be utilized in the field to improve nitrogen use efficiency in crops.

## Introduction

Nitrogen (N) is an essential nutrient for plants and is required in larger quantities than other soil nutrients for optimal growth (Clarkson and Hanson 1980; Marschner 1995). Plants primarily acquire N from the soil in the forms of nitrate and ammonium (Li et al. 2017; Miller and Cramer 2005; Owen and Jones 2001). Ammonium, which may be absorbed directly from soil or synthesized through the reduction of nitrate, is assimilated into glutamine. As autotrophs, plants synthesize all the N-containing organic compounds necessary for their growth and development from this assimilated N (Xu et al. 2012). The N-containing organic compounds include nucleic acids, amino acids, chlorophyll pigments, and phytohormones; however, a significant portion of the N assimilated by plants is allocated to producing the proteins involved in photosynthesis. Notably, ribulose 1,5-bisphosphate carboxylase/oxygenase accounts for 20–30% of leaf N (Makino 2011), underscoring the importance of a sufficient supply of N in maintaining photosynthesis. Consequently, photosynthetic activity declines in N-deficient environments, resulting in reduced crop biomass and lower yields (Mu and Chen 2021).

The essential role of N in plant growth means that high crop yields depend on a sufficient supply of N to crops. However, nitrogen limitation to primary production is believed to be widespread (Vitousek and Howarth 1991). Consistently, the remarkable increase in agricultural production over the 20th century has largely been attributed to the intensive use of N fertilizers (Erisman et al. 2008; Tilman et al. 2002), clarifying that N availability is a limiting factor for both crop growth and yield. Estimates suggest that N fertilizers are responsible for feeding at least 40% of the world’s population, based on the increases in crop yield that result from their application (Erisman et al. 2008; Fixen and West 2002; Smil 2002).

Ammonium and nitrate derived from N fertilizers are also absorbed and utilized by soil microorganisms, however, resulting in competition between plants and microorganisms for N sources. In terrestrial oxidative environments, ammonium is readily converted into nitrate, which is less likely to be adsorbed by soil particles and thus more likely to leach into the environment. As a result, it is estimated that crop species absorb less than 50% of the N fertilizer applied (Anas et al. 2020). An excess of N fertilizer is therefore often applied in the field to maximize crop yields, which results in significant diffusion of applied N into the surrounding environment, especially rivers and oceans. Furthermore, denitrification of nitrate by soil microorganisms releases nitrogen oxides (NOx) into the atmosphere; this is an indirect cause of acid rain. In rice paddies, soil microorganisms convert ammonia into nitrous oxide (N_2_O), a potent greenhouse gas (Anas et al. 2020); ammonia may also be released directly from paddy fields into the atmosphere, contributing further to the greenhouse effect. The use of N fertilizers in current agriculture has therefore become a major source of environmental stress (Robertson and Vitousek 2009).

Environmental pollution by N fertilizers does not occur exclusively in developed countries but is also prevalent in developing nations due to rises in N deposition resulting from socio-economic development (Zhu et al. 2025). Worldwide, there are increasing calls for reductions in the use of N fertilizers for reasons beyond environmental pollution, given that the production of nitrogen fertilizers is very energy-consuming and accounts for approximately 1.2% of the world’s total energy supply (Ghavam et al. 2021). Furthermore, the instability of N fertilizer prices has a significant impact on consistent crop production, especially in developing nations (Vos et al. 2025). The development of crops with high N use efficiency (NUE) offers a potential solution to the problems associated with N fertilizer use that would also enable sustainable agriculture. Molecular breeding programs aimed at creating such varieties, based on an understanding of the mechanisms regulating plant responses to N supply and N deficiency (collectively referred to as “the N response”), are thus critical to the field of plant biotechnology.

Our knowledge of the mechanisms controlling the N response has advanced significantly over the past decade, increasing the likelihood of developing crops with improved NUE. In this review, I summarize these recent developments in our understanding of these mechanisms, with a particular focus on the regulation of N response-related gene expression. I also explore the questions arising from this progress before discussing how these advances can be applied to enhance NUE in crops.

## Do nitrate and ammonium play a dual role in plants?

As plants are sessile organisms, they need to adapt to variations in N availability in an environment that may change temporally and spatially. To optimize their N acquisition and use in response to changes in N availability, plants adjust gene expression, metabolism, root structure, and uptake activity (Jia and von Wirén 2020; Kiba and Krapp 2016; Ueda et al. 2017). The main N sources for plants are nitrate and ammonium, each of which possesses distinctive characteristics. Nitrate is generally more abundant than ammonium in terrestrial aerobic environments but must be reduced to ammonium within plant cells to enable N assimilation. Since the process of nitrate reduction is energy-intensive (Guerrero et al. 1981), ammonium may appear to be the better N source in terms of energy expenditure; however, excess ammonium is toxic to plants (Liu and von Wirén 2017). It has been shown that excessive ammonium assimilation by plastidic glutamine synthetase (GS) results in proton accumulation and cellular acidification, while nitrate assimilation consumes protons and thereby mitigates this negative effect (Hachiya et al. 2021). Consistently, adding nitrate to the growth medium alleviates ammonium toxicity in Arabidopsis without lessening ammonium accumulation and organic acid depletion (Hachiya et al. 2012).

Uptake of nitrate and ammonium induces both shared and specific changes in plant morphology, metabolism, and gene expression (Hachiya and Sakakibara 2017; Jia and von Wirén 2020; Sato and Yanagisawa 2014; Ueda et al. 2017). Transcriptome analyses have clarified that supplying nitrate and ammonium triggers both common and distinctive changes in gene expression (Pélissier et al. 2024). Supplying N-starved plants with nitrate elevates the expression of genes related to nitrate assimilation, including genes encoding nitrate transporters, nitrite reductase, and nitrate reductase, as well as those associated with other metabolic pathways, such as the pentose phosphate and iron metabolism pathways (Wang et al. 2003). By contrast, supplying plants with ammonium activates genes related to defense and innate immune responses, the stress response, and the response to jasmonic acid, as well as several genes involved in N metabolism (Patterson et al. 2010).

A recent study identified NIN-like protein (NLP) transcription factors (TFs) as intracellular nitrate sensors that directly bind to nitrate in Arabidopsis (*Arabidopsis thaliana*) (Liu et al. 2022). Since NLP TFs are conserved across terrestrial plant species (Sakuraba et al. 2022), nitrate is probably a common signaling molecule. Plants that are grown in paddy fields generally prefer ammonium over nitrate as a N source. It is estimated, however, that rice (*Oryza sativa*) plants grown in paddy fields absorb more than 30% of their total N uptake from nitrate (Kirk and Kronzucker 2005), because ammonium is oxidized to nitrate by nitrifying bacteria in the partially aerobic environment near the root surface, where oxygen is supplied via aerenchyma (Colmer 2003; Rubinigg et al. 2002; Watanabe et al. 2013). Therefore, it is reasonable to conclude that nitrate plays a dual role in terrestrial plants, not only serving as an essential nutrient but also acting as a signal that conveys information regarding N availability and nutritional status.

By contrast, no specific sensors or receptors for ammonium have been identified, although there is circumstantial evidence suggesting that ammonium transporters (AMTs) may function as ammonium sensors (Liu and von Wirén 2017). This evidence includes the observed changes in ammonium-triggered lateral root branching in an *Arabidopsis* mutant that is defective in four root-expressed *AMT* genes (Lima et al. 2010) and the phosphorylation and subsequent inactivation of the AMT1;1 trimer in response to increased extracellular ammonium concentrations (Lanquar et al. 2009). However, it is also plausible that ammonium promotes N assimilation merely by acting as a N source whose uptake leads to a dramatic increase in the products of N assimilation, which, in turn, influences changes in gene expression and metabolism. Simultaneously, ammonium may trigger stress responses due to its toxicity, which is associated with changes in external pH (Pélissier et al. 2024; Rivero-Marcos et al. 2024). Therefore, it remains unclear whether ammonium also functions both as a nutrient and as a nutritional signal conveying information about N availability (Pélissier et al. 2024). Recently, a DNA region responsible for the ammonium supply-dependent activation of an Arabidopsis gene encoding NADH-dependent glutamine 2-oxoglutarate aminotransferase (GOGAT; glutamate synthase), known as *GLT1*, has been identified in its promoter region (Kojima et al. 2023). However, it has also been suggested that the upregulation of *GLT1* expression depends on ammonium assimilation rather than ammonium itself (Kojima et al. 2023). Therefore, further analysis is necessary to determine the exact role of this DNA element.

## The mechanism underlying nitrate signaling

It was proposed more than 30 years ago that nitrate is both a nutrient and a signal involved in N assimilation (Crawford 1995). The latter function of nitrate was confirmed in a study showing that the provision of nitrate upregulated the expression of N assimilation-related genes even in Arabidopsis mutants lacking nitrate reductase activity (Wang et al. 2004). Although the mechanism controlling nitrate-induced gene expression remained elusive for a considerable time, studies primarily conducted in Arabidopsis have led to remarkable advances in our understanding (Ruffel et al. 2025; Vidal et al. 2020; Wang Y et al. 2018). The first clue towards deciphering the mechanism came from an analysis that identified a nitrate-responsive *cis*-regulatory DNA element (NRE) in the promoter of *NITRITE REDUCTASE1* (*NIR1*) in Arabidopsis (Konishi and Yanagisawa 2010). This NRE is conserved across the promoters of nitrite reductase genes of various plant species (Konishi and Yanagisawa 2011a) (Table 1). Furthermore, closely related sequences found in the promoter regions and/or the neighborhoods of other nitrate-inducible genes involved in nitrate reduction also function as NREs (Table 2). These genes include *NRT2.1* and *NRT2.2*, which encode high-affinity nitrate transporters (Maeda et al. 2018), *NITRATE REDUCTASE 1* (*NIA1*), which encodes nitrate reductase (Konishi and Yanagisawa 2011b, 2013a), and *NITRITE TRANSPORTER 2;1* (*NITR2;1*) and *NITR2;2*, which encode nitrite transporters (Maeda et al. 2014).

**Table 1:** Table 1. Putative NLP-binding sites in the nitrite reductase gene promoters from various plant species.

Plant species	DNA sequence	Position
*Arabidopsis thaliana*	**TGACCCTT**TACATTGCTC**AAGAG**CT**C**	−94 to −69
*Spinacia oleracea*	**TGACCCTT**AACCATGTCC**AAGAG**T**CC**	−223 to −198
*Sorghum bicolor*	**TGGCTCTT**GGGGAGTTCA**AAAGGGCA**	−116 to −141
*Oryza sativa*	**TGGCCCTT**GGCGAATTCA**AAAGGGCA**	−166 to −191
*Oryza sativa*	**TGGCTCTT**GGCGAATTCA**AAAGGGCA**	−155 to −180
Consensus	TG(A/G)C(C/T)CTT-N_10_-AA(G/A)(A/G)GGCA	

The semi-palindromic sequences enabling NLP transcription factor binding are given with conserved nucleotides marked in bold. Nucleotide positions are numbered relative to the translation start site. Only the NLP-binding site in the Arabidopsis promoter has been experimentally identified. The accession numbers of the nucleotide sequences, which include the nitrite reductase gene promoter sequences from *Spinacia oleracea*, *Sorghum bicolor*, and *Oryza sativa*, are X17031, Sb04g034160, and Os01g0357100, respectively, in the GenBank or Gramene databases.

**Table 2:** Table 2. Locations of *cis*-elements bound by NLP transcription factors in Arabidopsis nitrate-responsive genes.

Gene	Location	DNA sequence	Position
*NRT2.1* ^1^	Promoter (proximal)	**C**A**ACCTTT**GGTGATAAGCG**AGAGACT**	−172 to −150
	(distal)	**AAGAGAC**A	−714 to −707
*NRT2.2* ^1^	Promoter (proximal)	**T**A**ACCTTT**GGGGATTAGCG**AGAGA**G**T**	−208 to −186
	(distal)	T**AGAGAC**A	−537 to −530
*NIA1* ^2^	Promoter	ACCCGTC**CCTTT**	>1.8 kb upstream ^3^
		CG**CC**AC**T**	>1.8 kb upstream ^3^
	3′ flanking region ^5^	**TGACCCTT**GTAAGCACGA**AA**C**A**CGT**T**	+5103 to +5081
*NIR1* ^2^	Promoter	**TGACCCTT**TACATTGCTC**AAGAG**CTC	−94 to −69
*NITR2;1* ^2^	Promoter	**TGACCTTT**CGTTTAATGG**AAGAGACT**	−1045 to −1003
*NITR2;2* ^2^	Promoter	**TGACCTTT**CGACTGATCA**AA**C**AGACT**	−591 to −549
*BT1* ^2^	Promoter	**CGACCCTT**GCCTCTTCCC**AAGAG**C**C**C	−1464 to −1506
*BT2* ^2^	Promoter	**CGACCTTT**GCCTCTTCCC**AAGAG**C**C**A	−1300 to −1342
*AO* ^1^	Promoter	**TG**G**CC**G**TT**	−354 to −347
*HB52* ^1^	Promoter	**TGACCC**C**T**	−500 to −493
*HB54* ^1^	untranslated region	**TGAC**T**CTTAAGAG**T**C**C	+252 to +266
*NIGT1.1* ^2, 4^	3′ flanking region	**TGACCCTT**ACCTTCTCCT**AA**AG**G**C**C**A	+2599 to +2641
*NIGT1.2* ^2, 4^	3′ flanking region	**TGACCCTT**CGCTTTGTCGT**A**AG**G**C**C**A	+2933 to +2891
*NIGT1.3* ^2, 4^	3′ flanking region	**TGACCTTT**CAGAGTCATA**AA**A**AG**C**CT**	+3228 to +3270
*NIGT1.4* ^2, 4^	3′ flanking region	**TGACCTTT**CACACAAGTT**AA**AG**GAC**A	+3270 to +3312
Consensus		(T/C)GACC(C/T)TT-N10-AAGAGACT	

Conserved nucleotides are shown in bold. ^1^ Nucleotide positions are numbered relative to the transcriptional start site. ^2^ Nucleotide positions are numbered relative to the translation start site. ^3^ The sequence is located more than 1.8 kb upstream of the transcription start site, although its exact position in the *NIA1* promoter is not given in the relevant references. ^4^ Although both nitrate-responsive *cis*-elements and NLP-binding sites have not been identified experimentally in the vicinity of these genes, they contain putative NLP-binding sites in their 3′ flanking regions. ^5^ The NRE sequence is located on the anti-sense strand.

**Figure 1:**
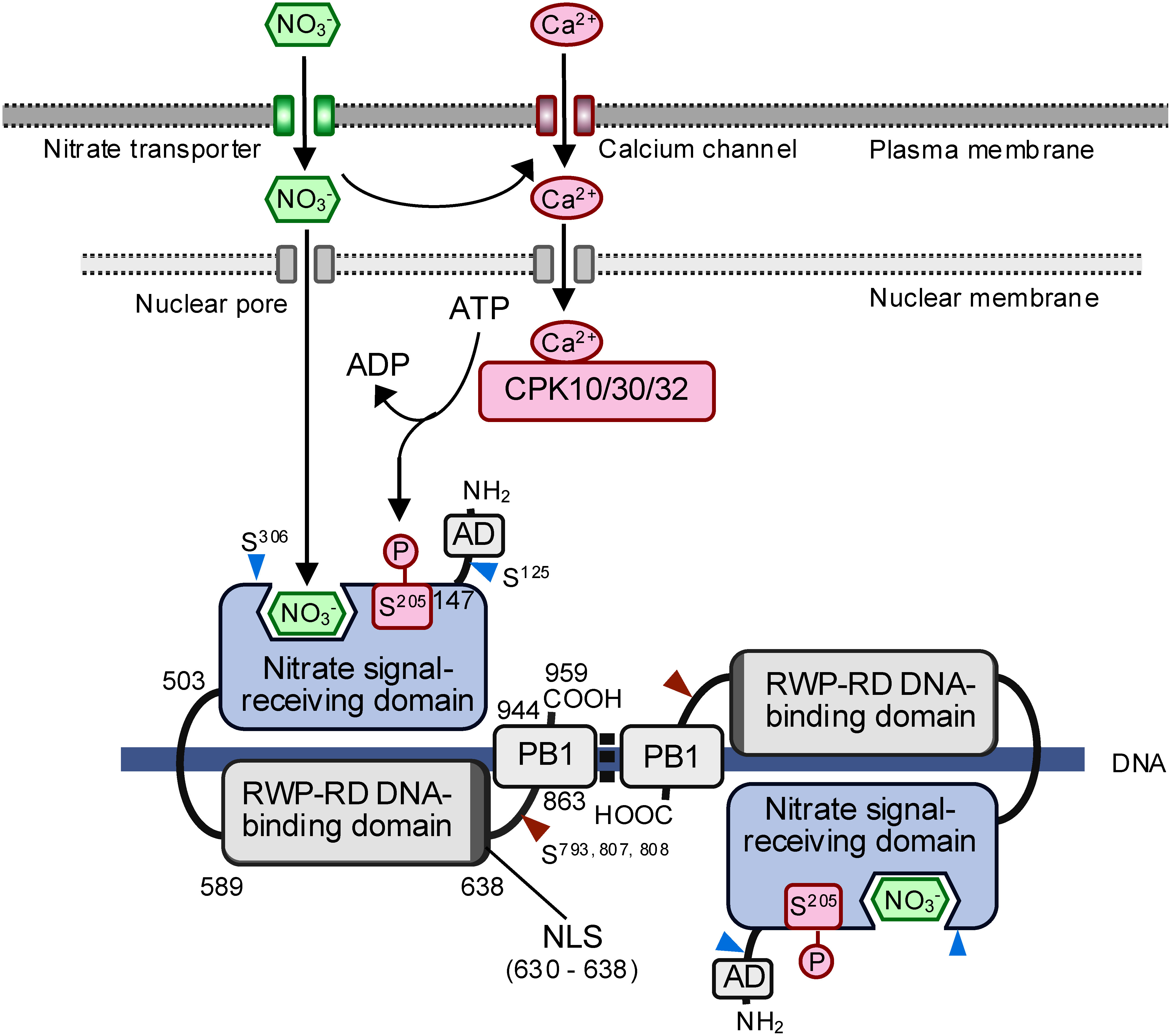
Figure 1. Hypothetical scheme illustrating the early stages of nitrate (NO_3_^−^) signaling. This scheme features Arabidopsis NIN-LIKE PROTEIN 7 (NLP7), the best-characterized NLP transcription factor (TF). Nitrate stimulates a unique and dynamic Ca^2+^ signature, which, in turn, activates the Ca^2+^-sensor protein kinases, CPK10, CPK30, and CPK32. In the cell nucleus, the activated CPKs phosphorylate a conserved Ser residue (Ser^205^ in NLP7) located in the nitrate signal-receiving domain of NLP TFs. The same domain also directly binds to nitrate. Activated NLP7 remains in the nucleus and binds to nitrate-responsive *cis*-elements (NREs) in the vicinity of nitrate-inducible target genes to enhance their transcription. NLP TFs form dimers via their PB1 domain, although the structural arrangement of each subunit has not yet been clarified. Brown arrowheads: Ser residues phosphorylated by CPK28; blue arrowheads: Ser residues phosphorylated by carbon deficiency-activated KIN10; NLS: nuclear localization signal; AD: transcriptional activation domain.

Subsequently, yeast one-hybrid screening revealed that NLP TFs bind directly to the NRE and promote transcription (Konishi and Yanagisawa 2013b). The NLP family is one of the two subfamilies of the RWP-RK DNA-binding domain-containing TF family (Chardin et al. 2014; Konishi and Yanagisawa 2014). The Arabidopsis genome encodes nine NLP TFs (NLP1–9) that possess the conserved domains for nitrate signal reception and DNA-binding, as well as the PB1 protein-protein interaction domain, which is responsible for homo and heterodimerization between NLPs (Guan et al. 2017; Konishi and Yanagisawa 2019) (Figure 1). These proteins also include a nuclear localization signal (NLS) that overlaps the RWP-RK DNA-binding domain (Liu et al. 2022). Additionally, a transcriptional activation domain has been identified at the N-terminus (amino terminus) of NLP7 (unpublished data). As the N-terminus amino acid sequences of the different NLP TFs vary (Suzuki et al. 2013), their transcriptional regulatory domains may also differ (Figure 1). All the Arabidopsis NLP TFs examined to date (NLP1, NLP3, NLP5, NLP6, NLP7, NLP8, and NLP9) are capable of binding to the NRE; this result is consistent with the highly conserved nature of their RWP-RK DNA-binding domains and indicates that members of this family bind to the NRE in a sequence-specific manner (Konishi and Yanagisawa 2013b; Yan et al. 2016). Although supplying nitrate does not induce expression of *NLP* genes or only slightly induces a few *NLP* genes (Konishi and Yanagisawa 2013b; Liu et al. 2017), nitrate activates NLP TFs post-transcriptionally, suggesting that these proteins are already present before nitrate signaling begins and can act as primary TFs regulating the expression of nitrate-responsive genes (Konishi and Yanagisawa 2013b). The nitrate signal-receiving domain in the N-terminal region of NLP TFs confers nitrate responsiveness upon an artificial TF that combines the nitrate signal-receiving domain, an NLS, and the DNA-binding domain from the bacterial LexA repressor, which indicates that the nitrate signal-receiving domain functions independently from other domains (Konishi and Yanagisawa 2013b). Two relevant studies also supported the role of the NLP family in nitrate-responsive gene expression. Since Arabidopsis *NLP7*-knockout mutants constitutively displayed several features typical of N-starved plants (Castaings et al. 2009), NLP7 was further characterized, revealing that nitrate regulates the localization of NLP7 to the nucleus via a nuclear retention mechanism (Marchive et al. 2013). A forward genetic screen of Arabidopsis mutants designed to identify *trans*-acting factors involved in nitrate responses identified an *nlp7* mutant deficient in nitrate responses, although further analyses of this mutant were not conducted (Wang et al. 2009).

Our current understanding that NLP TFs play a dual role in nitrate signaling results primarily from analyses of Arabidopsis NLP7. In the current model of the early stages of nitrate signaling (Figure 1), NLP TFs function as receptor-type TFs (Liu et al. 2022), as do nuclear thyroid hormone receptors in animal cells (Wu and Koenig 2000). They are thus responsible both for sensing intracellular nitrate concentration and regulating the expression of primary nitrate-responsive genes. Genome-wide analyses of relevant Arabidopsis mutants and transgenic lines support the view that NLP TFs are the primary TFs in nitrate signaling and regulate the expression of the majority of nitrate-responsive genes, either directly or through transcriptional cascades (Alvarez et al. 2020; Konishi and Yanagisawa 2014; Liu et al. 2022; Marchive et al. 2013). NLP TFs are activated post-translationally by the direct binding of nitrate (Liu et al. 2022) and the phosphorylation of a conserved serine (Ser) residue in the nitrate signal-receiving domain (Liu et al. 2017). The active forms of NLP TFs then interact with NREs located in the promoters or vicinity of their target genes, inducing their expression. The dissociation constant (*K*d) between NLP7 and nitrate is approximately 50 µM (Liu et al. 2022), which may be suitable for sensing intracellular nitrate that is also utilized as a N source. Additionally, it has been demonstrated that three amino acid residues in the nitrate signal-receiving domain of NLP7 (His-404, Leu-406, and Tyr-436) are crucial for nitrate binding, suggesting that this domain is also responsible for binding to nitrate (Liu et al. 2022). Meanwhile, it has also been shown that phosphorylation of the conserved Ser residues in NLP TFs (for example, Ser-205 in NLP7) is catalyzed by three calcium-dependent protein kinases, CPK10, CPK30, and CPK32. A corresponding nitrate-triggered Ca^2+^ signature has been detected in plant cells, and CPK10 and NLP7 directly interact in the nucleus in the presence of nitrate (Liu et al. 2017).

NLP TFs are probably present in all land plants and form a TF family that includes the legume-specific NODULE INCEPTION (NIN) proteins (Sakuraba et al. 2022). The first member of this family to be characterized was the NIN protein from *Lotus japonicus*, which plays an essential role in nodulation (Schauser et al. 1999). Owing to mutations within their nitrate signal-receiving domain, NIN proteins lack the conserved Ser residues required for nitrate-induced phosphorylation. Furthermore, unlike Leu-406 and Tyr-436, His-404 in NLP7 is not conserved in NIN proteins. Consequently, unlike NLP TFs, NIN TFs do not respond to nitrate but are constitutively active transcriptional activators that act independently of the presence or absence of nitrate (Suzuki et al. 2013). NIN proteins are conserved in legumes and form a subgroup of the NLP TF family that is distinct from other NLP TFs (Chardin et al. 2014; Sakuraba et al. 2022). NIN proteins in two legume species, *Medicago truncatula* and *Lotus japonicus*, have been shown to mediate the negative effect of nitrate on nodulation and form heterodimers with NLPs via their PB1 domain (Lin et al. 2018; Nishida et al. 2021). NIN is thus a variant NLP TF that has acquired a unique role in legumes but remains associated with nitrate signaling.

## Emerging questions concerning the nitrate signaling mechanism

Despite these remarkable advances in understanding the nitrate signaling mechanism, several questions remain unresolved. The first pertains to the initiation of the nitrate signaling process. Studies of NLP7 have identified three key steps in the post-translational activation of NLP TFs by nitrate: the nuclear retention of NLP TFs (Marchive et al. 2013), the phosphorylation of a conserved serine residue in the nitrate signal-receiving domain (Liu et al. 2017), and the direct binding of nitrate to NLP TFs (Liu et al. 2022). However, the relationships between these three steps remain unclear, and the sequence in which they occur has yet not been determined. Interestingly, although nitrate affects NLP7 accumulation in cell nuclei (Marchive et al. 2013), it does not affect the nuclear localization or accumulation of NLP8, a regulator of nitrate-promoted seed germination in Arabidopsis (Yan et al. 2016). Thus, nitrate may not universally regulate the nuclear retention of all NLP TFs; instead, the nitrate-dependent nuclear retention could be an ancillary mechanism that enhances the activity of NLP TFs by retaining activated NLP TFs in the nucleus. To understand the significance of enhanced nuclear retention more fully, as well as how to enhance it further, a systematic analysis of the nuclear retention of various NLP TFs is required. This first question is also associated with the mechanism by which the nitrate-receiving domain of NLP TFs regulates their nitrate-dependent activity. As noted previously, the calcium-dependent protein kinases CPK10, CPK30, and CPK32 phosphorylate Ser-205 of NLP7 in the nucleus in the presence of nitrate (Liu et al. 2017). However, these protein kinases do not function exclusively in nitrate signaling. CPK10 also enhances drought tolerance by affecting abscisic acid (ABA)-mediated regulation of stomatal closure and opening (Zou et al. 2010), CPK10 phosphorylates BOR1, a boron transporter, at the Ser-689 residue, enhancing plant tolerance to boron deficiency (Wang et al. 2024), and CPK32 is involved in controlling the polar growth of pollen tubes (Zhou et al. 2014). These observations pose the additional question of how NLP TFs are specifically phosphorylated by these three calcium-dependent protein kinases in response to nitrate. The initiation step of the nitrate signaling pathway therefore needs to be properly elucidated.

The second question concerns the relationship between NLP TF-mediated regulation and the function of NRT1.1 (also known as CHL1 and NPF6.3), which has also been proposed as a nitrate sensor (Ho et al. 2009). NRT1.1 is a unique transporter belonging to the NRT1/NPF family. Unlike other nitrate transporters, NRT1.1 exhibits either low or high affinity for nitrate depending on the phosphorylation state of Thr-101 (Liu and Tsay 2003) and thus is capable of nitrate uptake both under low (<1 mM) and high (>1 mM) nitrate conditions (Huang et al. 1996; Ye et al. 2019). The role of NRT1.1 as a nitrate sensor has been suggested through the characterization of two Arabidopsis mutants for *NRT1.1*, referred to as *chl1-5* and *chl1-9.* The *chl1-5* mutant is a deletion mutant that does not express the NRT1.1 protein (Tsay et al. 1993), while *chl1-9* expresses a mutant version of NRT1.1 (NRT1.1^P492L^) due to a point mutation. As a result, nitrate uptake was defective in *chl1-5*, whereas *chl1-9* exhibited a biphasic response for nitrate uptake, although both its high- and low-affinity nitrate uptake activities were significantly reduced in *chl1-9*. Furthermore, the nitrate-induced expression of *NRT2.1*, a typical nitrate-responsive gene that encodes a nitrate transporter, varied between these mutants; the expression was mostly diminished in *chl1-5*, whereas it remained unchanged in *chl1-9.* Thus, it can be concluded that nitrate sensing and uptake are decoupled in *chl1-9*, suggesting that NRT1.1 is a nitrate sensor that detects extracellular nitrate concentrations and undergoes phosphorylation in response (Ho et al. 2009). However, it was later demonstrated that the nitrate transport ability of NRT1.1^P492L^ might not lead to nitrate uptake into the cell because NRT1.1^P492L^ was not present at the plasma membrane, although there remains a possibility that NRT1.1^P492L^ affects nitrate uptake in a complicated manner (Bouguyon et al. 2015). Furthermore, it was found that, unlike *NRT2.1*, nitrate-induced rapid activation of several genes was similarly reduced in both *chl1-5* and *chl1-9* (Vidal et al. 2014). Therefore, the precise mechanism by which NRT1.1 functions as a nitrate sensor to regulate nitrate-responsive gene expression remains poorly understood, and the relationship between NLP TF-mediated regulation and the nitrate sensor function of NRT1.1 is currently unclear. Recent studies have begun to shed light on this question. Using the roots of Arabidopsis expressing aequorin, nitrate treatment was shown to transiently increase cytosolic Ca^2+^ concentration ([Ca^2+^]cyt), resulting in a peak that lasts approximately 10 s. This peak in [Ca^2+^]cyt was not observed in the *chl1-5* and *chl1-9* mutants (Riveras et al. 2015). Subsequently, a cyclic nucleotide-gated channel protein, CNGC15, and NRT1.1 were found to constitute a molecular switch that regulates calcium influx responsible for this nitrate-triggered peak in [Ca^2+^]cyt (Wang et al. 2021). Therefore, an increase in nitrate levels may enhance the calcium-channel activity of the NRT1.1-CNGC15 complex, generating the nitrate-induced influx of Ca^2+^ into the cell, which promotes the phosphorylation of NLP7 (Wang et al. 2021). Consistently, nitrate-induced levels of several nitrate-inducible genes were shown to be reduced in the *cngc15* (Wang et al. 2021). However, further analysis is necessary to clarify the relationship between NLP TF-mediated regulation and the nitrate sensor function NRT1.1, because it remains uncertain whether the gradually rising subcellular Ca^2+^ signature attributed to nitrate over a course of minutes, which was observed using a single-cell system in relation to the phosphorylation of NLP6 and NLP7 (Liu et al. 2017), is similarly abolished in *chl1-5*, *chl1-9*, and *cngc15* mutants. On the other hand, it has been shown that NLP7 directly regulates the expression of *NRT1.1* (Zhao et al. 2018).

The third question pertains to the recognition of target DNA sequences by NLP TFs. NLP6 and NLP7 recognize the semi-palindromic NRE sequence in the *NIR1* promoter. A comparison of *NIR1* promoter sequences across various plant species suggests that the consensus sequence for NLP6 and NLP7 binding is TG(A/G)C(C/T)CTT-N_10_-AA(G/A)(A/G)GGCA (Konishi and Yanagisawa 2014) (Table 1). The NLP7-binding sites identified in the promoters of various Arabidopsis nitrate-inducible genes, including *NITR2;1* and *NITR2;2* (Maeda et al. 2014) as well as *BT1* and *BT2* that encode proteins with unknown functions (Sato et al. 2017), are also semi-palindromic sequences that function as NREs, revising the consensus sequence for NLP7 binding (Table 2). As members of the NLP family form homo and heterodimers via the PB1 domain located at their C-terminus (carboxy terminus) (Konishi and Yanagisawa 2019; Lin et al. 2018; Nishida et al. 2021), it seems reasonable that NLP TFs bind to a semi-palindromic sequence (Figure 1). It is noteworthy, however, that only one of the two NLP7 binding sites in the promoters of *NRT2.1* and *NRT2.2* is a semi-palindromic NRE sequence, as the other contains only half of a semi-palindromic sequence (Maeda et al. 2018). Additionally, a highly degraded semi-palindromic sequence located in the 3′-flanking region of *NIA1* is an NRE sufficient for directing nitrate-inducible expression (Konishi and Yanagisawa 2011b, 2013a). Furthermore, the sequences of two NLP7-binding sites in the *NIA1* promoter, whose exact positions in the *NIA1* promoter region were not given in the relevant references (Guan et al. 2017; Wang et al. 2010), do not resemble those of other NREs (Table 2). A recent study showed that leghemoglobin genes from *M. truncatula* are regulated by more complicated NREs, termed double-NREs (dNREs) (Jiang et al. 2021). In dNREs, the second half of the first semi-palindrome NRE overlaps with the first half of the second NRE element (Jiang et al. 2021). These findings indicate that a deeper structural understanding of the NLP-NRE interaction is necessary to fully comprehend which DNA sequences are recognized by NLP TFs to select their target genes. Although the RWP-RK domain is a highly conserved DNA-binding domain (Chardin et al. 2014; Konishi and Yanagisawa 2014), it will be necessary to investigate the DNA-binding specificity of each NLP TF further, because each member regulates a different set of genes (Liu et al. 2022). In addition, heterodimerization between members of the NLP family (Konishi and Yanagisawa 2019; Lin et al. 2018; Nishida et al. 2021) may further complicate the pattern of DNA recognition by NLP TFs.

Fourthly, the roles of each member of the NLP family have not yet been clarified. Of the nine NLP TFs known from Arabidopsis, NLP7 plays the main role, and NLP6 an auxiliary role, in nitrate signaling during young seedling growth (Castaings et al. 2009; Cheng et al. 2023; Guan et al. 2017; Konishi and Yanagisawa 2013b). NLP2 has also been identified as a key player in nitrate signaling during vegetative growth (Konishi et al. 2021). Since NLP2, unlike NLP7, regulates genes involved in the oxidative pentose phosphate pathway, it has been proposed that, in response to nitrate availability, NLP2 integrates N assimilation with the supply of energy and carbon skeletons (Durand et al. 2023). NLP8 is responsible for nitrate-induced promotion of germination by increasing the expression of *CYP707A2* (Yan et al. 2016), a gene encoding an ABA catabolic enzyme (Kushiro et al. 2004). However, future studies are required to clarify the roles of the other NLPs.

Finally, recent advances in our understanding of nitrate signaling suggest the existence of several mechanisms linking the nitrate signaling pathway with other nutrient and stress signaling pathways, raising new questions concerning the details of these mechanisms. Carbon (C) deficiency-activated KIN10, the α-catalytic subunit of sucrose non-fermenting 1 (SNF1)-related kinase 1 (SnRK1), phosphorylates Ser-125 and Ser-306 in NLP7, which results in its cytoplasmic localization and degradation of NLP7. Thus, C deficiency-activated KIN10 is responsible for the inhibitory effects of C deficiency on the nitrate-dependent activation of NLP7 target genes as well as the nitrate-mediated promotion of growth (Wang et al. 2022). It is likely that regulation of NLP7 activity by KIN10 is involved in coordinating C and N metabolism, because the set of genes regulated by KIN-10 overlaps well with that regulated by nitrate (Wang et al. 2022). It is worth noting, however, that Ser-125 is located outside the conserved domains and this residue is not conserved in other NLP TFs (Figure 1). Although Ser-306 is located within the nitrate signal-receiving domain, it is also not well conserved, being present in only four of the nine NLP TFs found in Arabidopsis. This suggests that most NLP TFs are not under the control of KIN10. Considering that NLP7 plays a major role in nitrate signaling during vegetative growth, it is possible that some NLP TFs coordinate C and N metabolism via KIN10-mediated phosphorylation, but this mechanism should be examined in plant species beyond Arabidopsis. On the other hand, a recent study showed that cold shock rapidly activates the Ca^2+^-dependent activity of CPK28, leading to the phosphorylation of three Ser residues (Ser-793, Ser-807, and Ser-808) in NLP7, which are located in highly variable regions between the RWP-RK DNA-binding and PB1 domains (Figure 1). This phosphorylation facilitates the translocation of NLP7 from the cytosol to the nucleus in a Ca^2+^-dependent manner under cold stress. Given that NLP7 directly regulates the expression of a set of cold-responsive genes, this study proposed that CPK28 and NLP7 function as master regulators that integrate cold-evoked Ca^2+^ signal and transcriptional reprogramming (Ding et al. 2022). However, the role of the CPK28-NLP7 regulatory module is more difficult to understand than that of the KIN10-NLP7 regulatory module, because it is unlikely that cold stress enhances nitrate-responsive gene expression. Furthermore, it has been shown that CPK28 positively regulates nitrate uptake under N deprivation conditions by phosphorylating NRT2.1 (Yue et al. 2025). Therefore, the CPK28-NLP7 regulatory module may instead regulate cold stress-inducible gene expression independently of NLP7’s role in nitrate signaling. This question needs to be clarified in future research.

In summary, our understanding of both nitrate sensing and signaling and of primary nitrate-responsive gene expression has significantly advanced over the past decade, and now provides a basic framework for these mechanisms in plants. These recent advances, however, raise several new and thus far unanswered questions that should be addressed in further research.

## Metabolic and hormonal regulation and transcriptional cascades controlled by NLP-mediated nitrate signaling

Supplying plants with nitrate has major effects on many aspects of their physiology, including N metabolism, morphology, and flowering (Ruffel et al. 2025; Vidal et al. 2020; Wang Y et al. 2018). Nitrate directly or indirectly alters the expression levels of more than 1,000 genes to varying degrees and in various time-dependent manners (Varala et al. 2018; Wang et al. 2003). The recent identification of the direct targets of NLP TFs has greatly enhanced our understanding of how nitrate functions as a signaling molecule that regulates a wide range of plant physiological processes.

**Figure 2:**
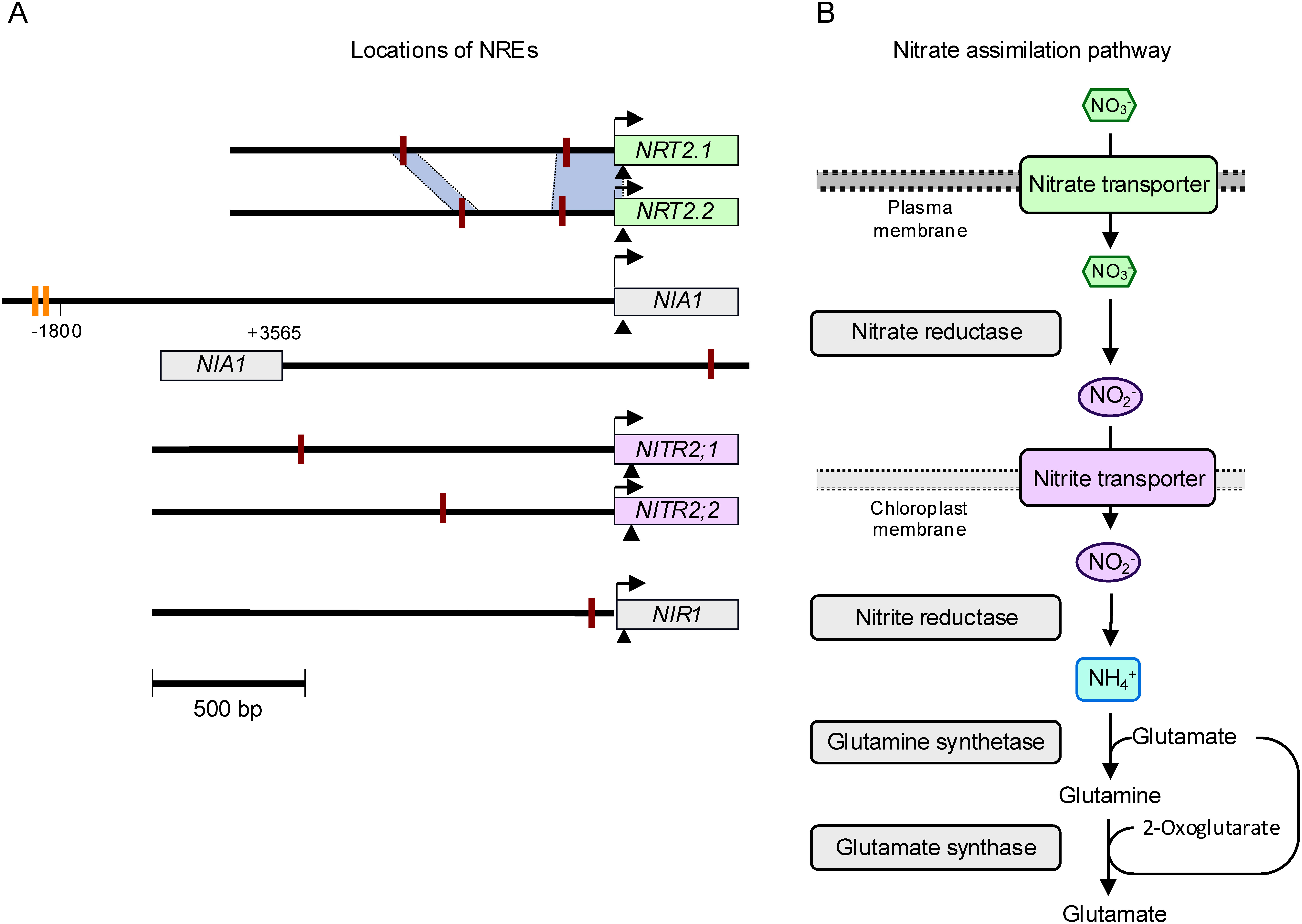
Figure 2. Regulation of the nitrate assimilation pathway by NLP TFs. (A) Locations of nitrate-responsive *cis*-regulatory DNA elements (NREs) in the vicinity of genes involved in nitrate reduction. *NRT2.1* and *NRT2.2* encode high-affinity nitrate transporters; *NIA1* encodes nitrate reductase; *NITR2;1* and *NITR2;2* encode nitrite transporters; *NIR1* encodes nitrite reductase. Red bars indicate the locations of NREs, while yellow bars indicate the locations of highly degraded semi-palindromic NREs in the 5′-upstream region of *NIA1*. Two homologous regions between the promoter sequences of *NRT2.1* and *NRT2.2*, which contain NREs, are indicated using two blue quadrilaterals. Black arrows: transcription start site; black arrowheads: translation start site. (B) Positions of the protein products from the *NRT2.1*, *NRT2.2*, *NIA1*, *NITR2;1*, *NITR2;2*, and *NIR1* genes in the nitrate assimilation pathway. Positions of glutamine synthetase and glutamate synthase (glutamine 2-oxoglutarate aminotransferase) involved in ammonium assimilation are also indicated.

A regulon is a group of genes regulated as a unit. The NLP7 regulon includes a set of N assimilation-related genes, as expected. The genes directly activated by NLP7 include those encoding high-affinity nitrate transporter (Maeda et al. 2018), nitrate reductase (Konishi and Yanagisawa 2013a), nitrite transporter responsible for the translocation of nitrite from the cytosol into chloroplasts (Maeda et al. 2014), and nitrite reductase (Konishi and Yanagisawa 2013b), thus NLP7 regulates all parts of the nitrate reduction pathway directly (Figure 2). Moreover, the identification of *L-ASPARTATE OXIDASE* (*AO*), a gene essential for de novo NAD^+^ biosynthesis, as a direct target of NLP7 revealed a new connection between nitrate signaling and metabolic regulation (Saito et al. 2022). The loss of nitrate responsiveness of the *AO* gene promoter has pronounced impacts on levels of TCA cycle-related metabolites, resulting in morphological changes and reduced growth; it does not, however, reduce nitrate reduction activity (Saito et al. 2022). These observations, together with the unique role of NLP2 in the oxidative pentose phosphate pathway (Durand et al. 2023), indicate that NLP TF activity is deeply involved in both C and N metabolism in plants.

The interaction between nitrate and cytokinin is well established. Nitrate signaling pathways utilize cytokinins as signaling molecules, especially *trans*-zeatin-type (tZ-type) cytokinin, which is translocated from the root to the shoot (Sakakibara et al. 2006). Targets of NLP7 include *CYP735A2*, which encodes cytokinin hydroxylase that catalyzes tZ-type cytokinin biosynthesis (Takei et al. 2004); this observation is consistent with the significant reduction in tZ-type cytokinin content in the Arabidopsis *nlp6 nlp7* double mutant (Maeda et al. 2018). Furthermore, grafting experiments have highlighted the importance of NLP7-mediated regulation of cytokinin biosynthesis and transport from the roots in regulating shoot developmental adaptation to nitrate availability (Abualia et al. 2022). These results, together with the role of NLP8 in degrading ABA (Yan et al. 2016), make clear that nitrate signaling is intimately linked with hormonal regulation through the NLP TF-regulated biosynthesis and degradation of plant hormones. Furthermore, a recent study indicated that nitrate suppresses ABA signaling through the physical interaction of NLP8 with two ABA signaling-associated TFs, ABSCISIC ACID INSENSITIVE 3 (ABI3) and ABI5 (Huang et al. 2025). This suggests that the NLP TF-mediated nitrate signaling also influences hormonal signaling.

NLP TFs also activate genes encoding other TFs, indicating that nitrate signaling may induce broad and complicated effects through transcriptional cascades. As an example, NLP7 binds to and activates the promoters of *HOMEOBOX PROTEIN52* (*HB52*) and *HB54*, two closely related homeodomain-leucine zipper (HD-Zip) class I TF genes. Their activation leads to elevated expression of *YELLOW VARIEGATED2* (*VAR2*), a target of HB52 and HB54 (Ariga et al. 2022). *VAR2* encodes the FtsH2 subunit of the chloroplast FtsH protease, which is responsible for the quality control of photodamaged thylakoid membrane proteins. Thus, genetic enhancement of the NLP7-HB52/54-VAR2 pathway improves light energy utilization under high light and low N conditions, identifying a connection between nitrate and light energy utilization (Ariga et al. 2022). Chip-seq analysis and detailed time-course analysis of nitrate-inducible gene expression patterns suggest that NLP7 directly regulates several TF genes, including *CIRCADIAN CLOCK ASSOCIATED 1* (*CCA1*), *CYCLING DOF FACTOR 1* (*CDF1*), and *TGACG-BINDING FACTOR 4* (*TGA4*) (Alvarez et al. 2020). A detailed analysis of the function of these TFs offers great promise for uncovering new roles for nitrate signaling and associated transcriptional cascades in plants.

## The NLP-NIGT1 regulatory module regulates responses to both nitrate supply and N deficiency

**Figure 3:**
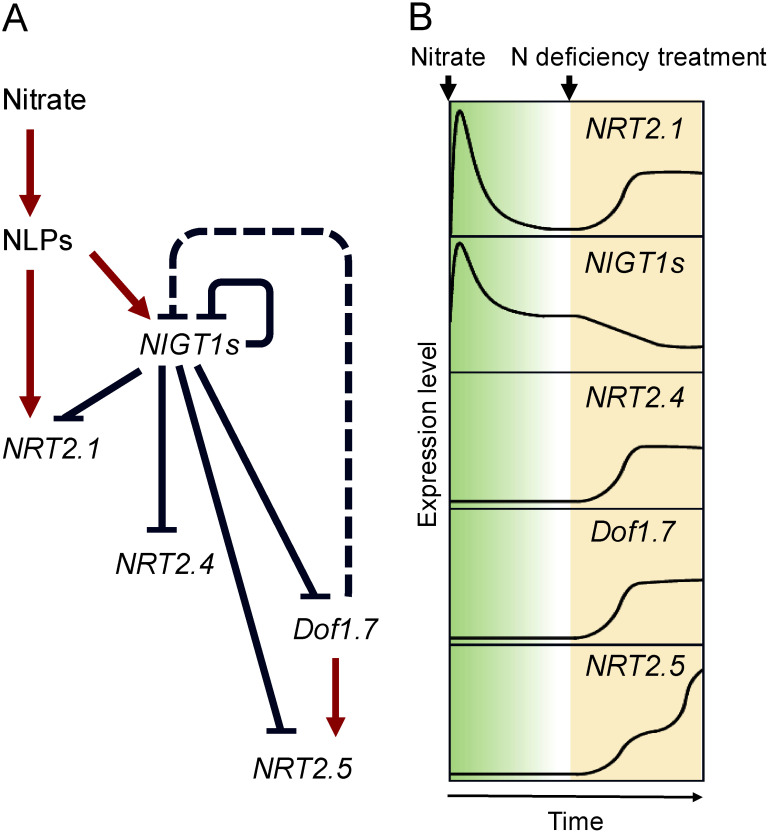
Figure 3. Nitrate-regulated dynamics of *NRT2* expression. (A) Schematic diagram of the gene regulatory network activated by nitrate. Red arrows indicate activation; blue blunt-ended lines indicate repression. This cascade includes the auto-repression of *NIGT1* genes as well as the indirect repression of *NIGT1.1* by Dof1.7. (B) Pattern diagram showing the time-dependent changes in expression of *NRT2.1*, *NRT2.4*, *NRT2.5*, *NIGT1*, and *Dof1.7* in response to nitrate and during N-deficiency treatment. Although the time-scale on the figure is arbitrary, the response to nitrate is a short-lived effect with gene expression peaking about an hour after the onset of nitrate supply. By contrast, the response to N starvation occurs over a far longer period of approximately a week.

Recent studies reveal that nitrate signaling is also involved in the N-deficiency response. In rice, *NITRATE-INDUCIBLE GARP-TYPE TRANSCRIPTIONAL REPRESSOR1* (*NIGT1*), which encodes a GARP/G2-like-type transcriptional repressor, is strongly and rapidly induced in response to nitrate (Sawaki et al. 2013). Its four Arabidopsis homologs, *AtNIGT1.1*, *AtNIGT1.2*, *AtNIGT1.3*, and *AtNIGT1.4*, are also nitrate-inducible genes regulated by NLP TF activity (Konishi and Yanagisawa 2014). AtNIGT1 functions as a transcriptional repressor of *NRT2.1*, which encodes a major high-affinity nitrate transporter. When N-starved Arabidopsis plants are supplied with nitrate, they activate NLP TFs that induce the expression of *AtNIGT1* genes, leading to the upregulation of common targets, such as *NRT2.1*, and their subsequent downregulation after a time-lag (Maeda et al. 2018) (Figure 3). This results in the regulation of nitrate-inducible genes via an incoherent feedforward loop (Goentoro et al. 2009). AtNIGT1 acts as a down-regulator within this loop, while NLP TFs act as transcription activators (Ueda and Yanagisawa 2019). This loop is more complex than a standard incoherent feedforward loop, however, due to auto-repression of *NIGT1* genes through the binding of NIGT1 proteins to their own promoters (Maeda et al. 2018; Sawaki et al. 2013). A combination of biology-based mathematical analysis and microplate-based expression monitoring revealed that complex regulation by NLP and NIGT TFs is responsible for the pattern of *NRT2.1* expression, which includes a transient peak in expression approximately 1 h after the initiation of nitrate treatment. Similar analyses revealed that the transcription module NLP-AtNIGT1 plays essential roles in stabilizing *NRT2.1* expression following perturbations in environmental nitrate concentrations and in generating appropriate changes in nitrate-inducible gene expression (Ueda and Yanagisawa 2023).

One-hybrid screening identified AtNIGT1 proteins as regulators that bind to the *cis*-regulatory elements responsible for the N deficiency-dependent activation of the *NRT2.4* and *NRT2.5* promoters (Kiba et al. 2018). When N-sufficient plants are exposed to N-deficient conditions, the expression of *AtNIGT1* genes decreases, leading to the expression of *NRT2.4* and *NRT2.5* (Figure 3). Thus, AtNIGT1 proteins also act as TFs that regulate gene expression in response to N deficiency (Kiba et al. 2018; Ueda and Yanagisawa 2019). Three *NRT2* genes, *NRT2.1*, *NRT2.4*, and *NRT2.5*, are responsible for >90% of nitrate uptake activity when N availability is low (Kiba and Krapp 2016; Lezhneva et al. 2014). As these three *NRT2* genes are under the control of NIGT1 proteins, this indicates that NIGT1 proteins are the critical regulators of N-deficiency responses.

The expression pattern of *NRT2.1* may appear confusing because its expression is induced both by nitrate and under conditions of N deficiency. The picture becomes clearer when the dynamic changes are understood as follows: supplying nitrate to N-starved plants induces *NRT2.1* expression due to NLP TF activity, but expression of *NRT2.1* is subsequently downregulated due to the activity of NIGT1 proteins. By contrast, when plants undergo a transition from N-sufficient to N-deficient conditions, NIGT1 activity declines due to the reduction in the nitrate content and this induces *NRT2.1* expression (Figure 3). Therefore, the increase and decrease in intracellular nitrate concentration are responsible for, respectively, the nitrate response and the N-deficiency response in gene expression. The nitrate response and the N-deficiency response can be viewed as two sides of the same coin; they are different outcomes from the same mechanism that are regulated by two antagonistic TF families, the NLP and NIGT1 families (Figure 3).

Genes that are both nitrate-inducible and N deficiency-inducible are antagonistically regulated by NLP activity and NIGT1 activity. This group of genes includes not only *NRT2.1* but also *CYP735A2* and *CYP707A*, which encodes ABA 8′-hydroxylase involved in ABA degradation (Kushiro et al. 2004). This highlights the regulatory links between response to N availability and hormonal regulation (Maeda et al. 2018). By contrast, some genes that are specifically regulated by NIGT1 activity, including those that, like *NRT2.4* and *NRT2.5*, are induced only under conditions of N deficiency. In Arabidopsis, NIGT1 also suppresses the expression of *Dof1.7* gene, which encodes a transcriptional activator of *NRT2.5* but not of *NRT2.4* (Zhuo et al. 2024). Under N-deficient conditions, passive transcriptional activation caused by decreased expression of *NIGT1* genes may therefore lead to transcriptional activation through the action of Dof1.7. In Arabidopsis, the NLP-NIGT1 regulatory module acts within the wider gene regulatory network and is responsible for time-dependent, multilayered transcriptional regulation of the three *NRT2* genes (Figure 3). This finding suggests that the NLP-NIGT1 regulatory module produces complicated regulatory responses to N deficiency. Although the complex phylogenetic relationships between the Dof TFs present in different plant species (Moreno-Risueno et al. 2007; Shigyo et al. 2007) make it difficult to identify orthologs of Arabidopsis Dof1.7, it is possible that homologs of Dof1.7 or other TFs play a similar role in the intricate regulation of *NRT2* genes via the NLP-NIGT1 regulatory module in other plant species.

A 3-day N starvation treatment reduced nitrate concentrations in Arabidopsis seedlings to levels below detection limits due to ongoing nitrate assimilation (Saito et al. 2022). Furthermore, the NRE-mediated activation of gene expression exhibited a diurnal pattern, likely responding to fluctuations in intracellular nitrate concentration due to nitrate assimilation (Konishi and Yanagisawa 2011a). These findings suggest that decreases in intracellular nitrate concentrations during N deficiency diminish the NLP-dependent activation of *NIGT1* genes, resulting in lower expression levels of *NIGT1* genes. Consequently, significantly reduced intracellular nitrate levels may trigger N deficiency responses. However, the responses of *NIGT1* genes to urea or glutamine application were complex in seedlings growing in a medium lacking both nitrate and ammonium (Kiba et al. 2018). Gene expression analysis conducted on whole seedlings indicated that, similar to nitrate, the application of urea or glutamine suppressed the reductions in expression levels of *NIGT1.1* and *NIGT1.2*, which are expressed in both shoots and roots (Maeda et al. 2018). By contrast, the application of urea or glutamine did not prevent the decrease in expression of *NIGT1.4*, which is expressed only in roots. Since nitrate assimilation primarily occurs in shoots (Scheurwater et al. 2002), this complexity can be explained by the following hypothesis: nitrate transport from roots to shoots continues irrespective of urea or glutamine supplementation, whereas the activity of nitrate assimilation in the shoots decreases with such supplementation, preventing reductions in nitrate concentration. This hypothesis needs careful evaluation in the future due to the more complex responses of *NIGT1.3* expression and the potential regulation by other N deficiency signals. However, significantly reduced intracellular nitrate levels may act as one of the signals regulating N deficiency responses. In addition to the regulation attributed to decreases in nitrate concentration, other mechanisms may also regulate responses to N deficiency. Levels of glutamine, the primary product of N assimilation in the GS/GOGAT cycle (Figure 2B), rapidly change in response to both N supply and N deprivation treatments, which suggests glutamine may be a signal of N-deficiency status (Kan et al. 2015; Lee et al. 2023). Despite this possibility, the signals of N deficiency, other than decreases in nitrate levels, remain poorly understood, as do the mechanisms that transduce N-deficiency signals into physiological responses. Despite the paucity of information on N-deficiency signals, *O. sativa HRS1 HOMOLOG3* (*OsHHO3*) plays a critical role in regulating responses to N deficiency in rice, independently of nitrate signaling. NIGT1 and HHO proteins make up a subfamily of the GARP-type TF family, which is called the NIGT1/HHO subfamily, as they have high similarity in their amino acid sequences and domain structures (Ohama and Yanagisawa 2024; Safi et al. 2017). The proteins from this subfamily form both homo and heterodimers (Ueda et al. 2020a). Despite these similarities, the proteins from the NIGT1/HHO subfamily are classified as either NIGT1 proteins encoded by nitrate-inducible genes or as HHO proteins encoded by nitrate-non-inducible genes (Ueda et al. 2020b). Co-expression analysis and machine learning-based pathway inference were conducted to explore the gene regulatory network required for the response to N deficiency in rice, and the results identified four TFs, including OsHHO3 and OsHHO4, as key regulators that act within the network in response to N deficiency (Ueda et al. 2020b). OsHHO3 is a transcriptional repressor of three critical AMT genes, *AMT1;1*, *AMT1;2*, and *AMT1;3*, which are responsible for most ammonium uptake activity in low ammonium concentration environments (Konishi and Ma 2021); it thus negatively regulates ammonium uptake activity (Liu et al. 2023). N deficiency leads to decreases in *OsHHO3* expression and, consequently, increases in ammonium uptake activity, maintaining rice growth in N-deficient environments (Liu et al. 2023). These findings suggest that N-deficiency responses are regulated by both nitrate-dependent and nitrate-independent mechanisms, further highlighting the critical roles of NIGT1/HHO family proteins in these regulatory processes. However, several key questions remain, including which molecule(s) functions as a signal of N deficiency, and how does N deficiency reduce the expression of *OsHHO3*?

## Biotechnology for improving NUE

Defining NUE in agriculture is complex. Previous reviews have offered several definitions (Good et al. 2004; Xu et al. 2012), some of which are based on total biomass, while others focus on crop yield. Although there is a strong positive relationship between total biomass and crop yield, they are not completely proportional. It is also necessary to distinguish NUE per area and NUE per plant. Furthermore, plant NUE is also complex and involves several factors that necessitate individual discussion, such as N uptake efficiency (NUpE), N utilization (assimilation) efficiency (NUtE), and N physiological use efficiency (NpUE) (Xu et al. 2012). These complexities mean there are multiple possible approaches to improving NUE in an agricultural context that depend on the specific traits and crops involved. Nevertheless, because all agricultural traits ultimately rely on plants receiving sufficient N, enhancing N uptake and assimilation is a general strategy for improving NUE in agriculturally valuable crops. In this context, the identification of the master regulators controlling N acquisition and assimilation and their modification via genetic engineering techniques are practical approaches to improving NUE in a wide range of crops. Identifying the TFs that synchronously regulate N assimilation-related genes is particularly important (Ueda and Yanagisawa 2018).

Strong expression of the maize (*Zea mays*) transcriptional activator Dof1, which is presumably involved in organic acid metabolism (Yanagisawa 2000; Yanagisawa and Sheen 1998), enhances growth under N-deficient environments of Arabidopsis (Yanagisawa et al. 2004), rice (Kurai et al. 2011), wheat (*Triticum aestivum*), and sorghum (*Sorghum bicolor*) (Peña et al. 2017), possibly by promoting production of the carbon skeletons necessary for N assimilation (Yanagisawa et al. 2004). This success contrasts with previous studies in which overexpression of genes encoding nitrate transporters or enzymes involved in N assimilation barely improved growth under N-deficient conditions (Good et al. 2004; McAllister et al. 2012; Xu et al. 2012), although subsequent studies indicated that overexpression of the dual nitrate transporter *NRT1.1* improves growth and grain yields in rice (Wang W et al. 2018) and growth in Arabidopsis (Sakuraba et al. 2021). Similarly, overexpression of *NLP7* enhances growth in Arabidopsis (Yu et al. 2016) and overexpression of *OsNLP1*, *OsNLP3*, and *OsNLP4* improves growth and grain yields in rice (Alfatih et al. 2020; Wu et al. 2021; Zhang et al. 2022). Since NLP TFs are master regulators that govern nitrate uptake, nitrate reduction, and ammonium assimilation, these findings are presumably as expected; however, since NLP TF activity is enhanced by nitrate-dependent post-translational modifications (Liu et al. 2017, 2022), further studies are needed to clarify why overexpression of NLP TFs enhances growth under N-deficient conditions.

Recent research suggests that, in addition to the overexpression of particular TF genes, genome editing of target genes can lead to improved NUE in crops. N-deficiency responses are negatively regulated by transcriptional repressors from the NIGT1/HHO family in Arabidopsis (Kiba et al. 2018) and rice (Ueda et al. 2020b), and the disruption of several *NIGT1*/*HHO* genes results in improved growth in N-deficient environments. In Arabidopsis, N deficiency leads to decreased expression of *AtNIGT1* genes, which in turn induces the expression of *NRT2* genes, resulting in increased nitrate uptake activity. Similarly, in rice, N deficiency leads to reduced expression of *OsHHO3*, which in turn induces the expression of *AMT1* genes, resulting in increased ammonium uptake activity in low ammonium concentration environments. Expression of *AtNIGT1* and *OsHHO3* genes persists in N-deficient environments, however, and thus the complete removal of their transcripts through gene knockout techniques, such as T-DNA insertion or Cas9-mediated genome editing, improves growth in N-deficient environments (Kiba et al. 2018; Liu et al. 2023; Safi et al. 2021). Since negative regulation by the NIGT1/HHO transcriptional repressor family plays an essential role in regulating the acquisition of N in monocots and dicots, genome editing approaches that target the NIGT1/HHO transcriptional repressor family may enhance N uptake activity and improve NUE in various crop plants. It is worth noting that OsHHO3 was identified as a key regulator based on natural variation in various rice cultivars (Liu et al. 2023; Ueda et al. 2020b). Thus, it should be possible to identify elite alleles of *OsHHO3*, suggesting that the *NIGT1*/*HHO* transcriptional repressor gene family may be a useful target in traditional crossbreeding programs.

In summary, recent achievements indicate that understanding the mechanisms regulating responses to N availability offers opportunities to develop crops with enhanced NUE.

## Concluding remarks and future perspectives

Our understanding of the mechanisms underlying plant responses to N availability has dramatically advanced following the identification of the key regulators involved. Furthermore, biotechnological trials suggest that NUE can be enhanced in crop plants by overexpressing the appropriate TFs. Importantly, recent studies have also revealed that the transcriptomic changes required for adaptation to N-deficient environments are driven, at least in part, by negative regulation by transcriptional repressors. This means that inactivating specific TF genes through CRISPR-Cas9-mediated genome editing could be utilized as a molecular breeding technique to enhance NUE in various crop species.

Despite these recent advances, many questions regarding the mechanisms regulating responses to N availability remain, especially concerning how adaptation to N-deficient environments is facilitated. However, a thorough understanding of the mechanisms that control plant responses to N deficiency is critical. As the benefit of improved NUE can be significantly limited by other factors that influence growth, such as light, temperature, or limited availability of other nutrients, it may be more productive to enhance NUE under N-limiting conditions than in environments with an excess of N. Recent studies have revealed that a decrease in intracellular nitrate concentration serves as a signal of N deficiency. It is likely, however, that plants possess additional ways of signaling N deficiency and thus, in order to fully comprehend the mechanisms involved in N-deficiency responses, a key focus of future work should be the identification of all N-deficiency signals. Further research into these mechanisms will provide new opportunities for improving NUE in crop plants grown in N-deficient environments, leading ultimately to an increase in their global yield.

## Abbreviations

ABA: abscisic acid
AMT: ammonium transporter
*AO*: *L-ASPARTATE OXIDASE*
C: carbon
*CCA1*: *CIRCADIAN CLOCK ASSOCIATED1*
*CDF1*: *CYCLING DOF FACTOR1*
GOGAT: glutamine 2-oxoglutarate aminotransferase
GS: glutamine synthetase
HB52: HOMEOBOX PROTEIN52
HD-Zip: homeodomain-leucine zipper
N: nitrogen
*NIA1*: *NITRATE REDUCTASE1*
*NIGT1*: *NITRATE-INDUCIBLE GARP-TYPE TRANSCRIPTIONAL REPRESSOR1*
NIN: NODULE INCEPTION
*NIR*: *NITRITE REDUCTASE*
*NITR2;1*: *NITRITE TRANSPORTER2;1*
NLP: NIN-like protein
NLS: nuclear localization signal
NRE: nitrate-responsive *cis*-regulatory DNA element
NRT: nitrate transporter
NUE: nitrogen use efficiency
TF: transcription factor
*TGA4*: *TGACG-BINDING FACTOR4*
tZ: *trans*-zeatin
